# Revising the WHO Essential Medicines List for paediatric rheumatology update

**DOI:** 10.1186/s12969-022-00752-0

**Published:** 2022-10-14

**Authors:** Waheba Slamang, Nicola Smith, Chris Scott, Helen Foster, on behalf of the Paediatric Global MSK Task Force

**Affiliations:** 1 PReS Global Musculoskeletal Task Force Research Fellow, University of Cape Town, Cape Town, South Africa; 2 Paediatric Rheumatology, University of Cape Town, Cape Town, South Africa; 3 Translational and Clinical Research Institute, Newcastle University, Newcastle upon Tyne, UK; 4 Population and Health Sciences Institute, Newcastle University, Newcastle Upon Tyne, UK

Dear Editor,

As the current World Health Organisation (WHO) Essential Medicines List (EML) for ‘Joint diseases in children’ does not reflect current best practice [1], the Paediatric Global Musculoskeletal Task Force (TF) 2021 survey [2] worked to identify ‘Essential’ medicines for rheumatic diseases, which informed our application to the WHO in 2021. With feedback from the WHO (to give more information about the use of these medicines in clinical practice), a further revised application to the WHO is planned for late 2022 and will focus on the medicines primarily used in JIA.

Healthcare professionals working in paediatric rheumatology and members of the TF were invited to participate in an anonymous online survey to update opinion about medicines to be included in the EML for JIA, and to identify challenges to their access, availability, administration, and safety.

We had 173 respondents from 46 countries across all continents, median years of clinical practice 10 years (range 0.5-35) including: paediatric rheumatologists (*n*=118); nurses/nurse practitioners (*n*=21); trainees in adult or paediatric rheumatology (*n*=14); and general paediatricians (*n*=11). Survey data were analysed with descriptive statistics.

The most important medicines to be included in the WHO EML for JIA are listed in Table 1. The *availability* of subcutaneous (*n*=107/173; 62%), intravenous (*n*=94/173; 54%), and intra-articular medicines (*n*=83/173; 48%), as well as the *affordability* of subcutaneous (*n*=111/173; 64%) and intravenous medicines (*n*=103/173; 60%), were identified as important factors limiting delivery of care.Timely access to day-case facilities (including general anaesthesia/sedation and availability of imaging to perform intra-articular injections), and geographic challenges (e.g. patients home being remote from the infusion centre), were additional limiting factors. Most responders reported the procedures for intra-articular injections (*n* = 138/171; 81%), subcutaneous injections (*n* = 123/173; 71%), and intravenous injections (*n* = 140/173; 81%) to be always available or available most of the time.

**Table 1:** Medicines to be included in the WHO EML for JIA (those selected as most important are underlined)

Medication	N (%)	Medication	N (%)
Methotrexate	173 (100%)	TNF Inhibitor	168 (97%)
−Methotrexate	−143 (83%)	−Adalimumab	−152 (88%)
−Methotrexate	−147 (85%)	−Etanercept	−115 (66%)
(Subcutaneous)		−Infliximab	−77 (45%)
Sulphasalazine	55 (32%)	IL6 Inhibitor	151 (87%)
		−Tocilizumab (Intravenous)	−121 (70%)
		−Tocilizumab (Subcutaneous)	−90 (52%)
Hydroxychloroquine	77 (45%)	IL1 Inhibitor	123 (71%)
		−Anakinra	−120 (69%)
		−Rilonacept	−4 (2%)
		−Canakinumab	−31 (18%)
Intra-articular Steroids	157 (91%)	Abatacept	39 (23%)
−Triamcinolone Hexacetonide	−134 (77%)		
−Triamcinolone Acetonide	−35 (30%)		
Methylprednisolone Acetate	−32 (19%)		
Prednisolone	150 (87%)	Rituximab	75 (43%)
Methylprednisolone (Intravenous)	123 (71%)	Tofacitinib	54 (31%)
Azathioprine	45 (26%)	Baricitinib	19 (11%)
Ciclosporin	33 (19%)	Leflunomide	2 (1%)
**Total N 173**			

Our survey demonstrates that the main barrier to these medicines being used in clinical practice is their availability and affordability rather than the availability of personnel to perform these procedures or concerns about procedure complication such as infection.

These survey data are in line with the previous 2021 survey in terms of the medicines considered most important for inclusion in the EML. The survey data will support our revised TF application in 2022 for medicines deemed to be ‘most essential’ in the treatment of JIA i.e. intra-articular steroids (*triamcinolone hexacetonide as the medicine of choice*), an IL1 inhibitor (*anakinra as the medicine of choice*) and *tocilizumab*, in addition to methotrexate and TNF inhibitors already listed in the WHO EML. The provision of this range of medicines in the WHO EML will facilitate their improved access, availability and affordability, to enable standard care in many more countries around the world.

## Data Availability

All data generated or analysed during this study are included in this published article (and its supplementary information files).

## Abbreviations

EML: Essential Medicines List JIA Juvenile Idiopathic Arthritis
TF: Paediatric Global Musculoskeletal Taskforce
TNF: Tumour Necrosis Factor
WHO: World Health Organisation
